# Integrative prognostic analysis of tumor–infiltrating lymphocytes, CD8, CD20, programmed cell death-ligand 1, and tertiary lymphoid structures in patients with early-stage triple-negative breast cancer who did not receive adjuvant chemotherapy

**DOI:** 10.1007/s10549-022-06787-x

**Published:** 2022-11-16

**Authors:** Shu Yazaki, Tatsunori Shimoi, Masayuki Yoshida, Hitomi Sumiyoshi-Okuma, Motoko Arakaki, Ayumi Saito, Shosuke Kita, Kasumi Yamamoto, Yuki Kojima, Tadaaki Nishikawa, Maki Tanioka, Kazuki Sudo, Emi Noguchi, Takeshi Murata, Sho Shiino, Shin Takayama, Akihiko Suto, Yuichiro Ohe, Yasuhiro Fujiwara, Kan Yonemori

**Affiliations:** 1 Department of Medical Oncology, National Cancer Center Hospital, 5–1–1, Tsukiji, Chuo–ku, Tokyo, 104–0045 Japan; 2 Cancer Medicine, Jikei University Graduate School of Medicine, Tokyo, Japan; 3 Department of Diagnostic Pathology, National Cancer Center Hospital, Tokyo, Japan; 4 Department of Breast Surgery, National Cancer Center Hospital, Tokyo, Japan; 5 Department of Thoracic Oncology, National Cancer Center Hospital, Tokyo, Japan

**Keywords:** Tumor-infiltrating lymphocytes, Programmed cell death-ligand 1, Triple-negative breast cancer, Prognosis, Chemotherapy

## Abstract

**Purpose:**

Stromal tumor-infiltrating lymphocytes (TILs) are independent prognostic factors in systemically untreated early-stage triple-negative breast cancer (TNBC). Other immune biomarkers including CD8, CD20, programmed cell death-ligand 1 (PD-L1), and tertiary lymphoid structures (TLS) are also reported to be associated with prognosis. However, whether combining other immune biomarkers with TILs would allow for further prognostic stratification is unknown.

**Methods:**

We retrospectively analyzed 125 patients with early-stage TNBC not receiving perioperative chemotherapy. Stromal TILs and TLS were evaluated on hematoxylin–eosin slides. PD-L1 expression was evaluated using the SP142 assay. CD8 and CD20 were assessed by immunohistochemistry and counted by digital pathology.

**Results:**

Immune biomarker levels were positively correlated (*p* < 0.001). Adding CD8 and PD-L1 to multivariable analysis including clinicopathological factors (stage and histological grade) and TILs significantly improved the prognostic model (likelihood ratio χ^2^ = 9.24, *p* = 0.01). In Cox regression analysis, high CD8 was significantly associated with better prognosis [hazard ratio (HR) 0.69, 95% confidence interval (CI) 0.48–0.98, *p* = 0.04], and PD-L1 positivity was significantly associated with worse prognosis (HR 4.33, 95%CI 1.57–11.99, *p* = 0.005). Patients with high CD8/PD–L1 (–) tumors had the most favorable prognosis [5 year invasive disease-free survival (iDFS), 100%], while patients with low CD8/PD-L1( +) tumors had the worst prognosis (5 year iDFS, 33.3%).

**Conclusion:**

CD8 and PD-L1 levels add prognostic information beyond TILs for early-stage TNBC not receiving perioperative chemotherapy. CD8–positive T cells and PD-L1 may be useful for prognostic stratification and in designing future clinical trials of TNBC.

**Supplementary Information:**

The online version contains supplementary material available at 10.1007/s10549-022-06787-x.

## Introduction

Triple-negative breast cancer (TNBC) accounts for 10–15% of breast cancer cases and is a subtype characterized by the lack of estrogen receptor (ER), progesterone receptor (PR), and human epidermal growth factor receptor 2 (HER2) expression/amplification [1]. Since TNBC is an aggressive disease and is associated with a worse prognosis than other subtypes [2], nearly all patients with early-stage TNBC are recommended to receive perioperative chemotherapy to prevent relapse [3]. Although TNBC patients with small tumor size (< 1 cm) and negative lymph nodes have a relatively good prognosis according to observational studies [4], a population that does not require perioperative chemotherapy has not been identified. Identifying populations with particularly favorable prognoses without perioperative chemotherapy may lead to the selection of patients who can be omitted from chemotherapy.

Tumor-infiltrating lymphocytes (TILs) are mononuclear immune cells within tumor tissue [5]. TILs have been reported to be favorable prognostic factors in many types of cancer [6]. There have been previous reports that TILs levels are associated with response to neoadjuvant chemotherapy in early-stage TNBC [7–9]. Moreover, stromal TILs are strong prognostic factors in early-stage TNBC with or without perioperative chemotherapy and provide additional prognostic information beyond TNM staging [9–12]. Although the importance of TILs as prognostic biomarkers have been included in several international guidelines for early-stage disease [3, 13], a more detailed characterization of the tumor immune microenvironment may be useful for further prognostic stratification.

CD8^+^ and CD20^+^ lymphocytes are the major components of TILs, and both are associated with a favorable prognosis in early-stage TNBC [14, 15]. The programmed cell-death ligand 1 (PD-L1) and programmed cell death receptor 1 (PD-1) axis are key immune evasion mechanisms. However, the prognostic value of PD-L1 expression in TNBC is still unclear [16]. PD-L1 expression in immune cells was correlated with high–risk clinicopathological features in early TNBC [17]. The tertiary lymphoid structure (TLS) resembles a secondary lymphoid organ, and its functions include the production of antigen-specific T cells and memory B cells [5, 18]. TLS has also been reported as a favorable prognostic factor in many cancer types, including TNBC [6, 19]. Therefore, the evaluation of CD8, CD20, PD-L1, and TLS in combination with TILs may provide further understanding of host tumor immunity and prognostic information. However, few studies have comprehensively evaluated these immune biomarkers in early-stage TNBC, and data on patients not treated with perioperative chemotherapy are limited.

In this study, we aimed to evaluate the prognostic significance of CD8, CD20, PD-L1, and TLS in combination with TILs in patients with TNBC not receiving adjuvant chemotherapy.

## Material and methods

### Study population

We identified patients with TNBC who underwent curative surgery and did not receive neoadjuvant or adjuvant chemotherapy at the National Cancer Center Hospital (Tokyo, Japan) between January 2001 and December 2015. We recommended perioperative chemotherapy for nearly all the patients with TNBC except for those with small tumor size (< 1 cm). We included patients who had not received chemotherapy for any reason (including advanced age, comorbidities, and patient preference), even those that were recommended to receive chemotherapy. We excluded patients with unavailable or insufficient tumor tissue. TNBC was defined as ER, PR, and HER2 negativity. ER and PR negativity was defined as < 10% immunohistochemical (IHC) stained tumor cells. HER2 negativity was defined as IHC 0/1 or 2 + and fluorescence in situ hybridization (FISH) was not amplified.

### Histopathological evaluation

Whole tumor sections of the surgical specimens were evaluated. TILs and TLS were assessed on hematoxylin and eosin (H&E) stained slides by two investigators (M.Y. and S.Y.). Stromal TILs were evaluated according to the International Immuno–Oncology Biomarker Working Group guidelines [20]. TILs were scored using semicontinuous (10% increments) methods and grouped into two categories: low (< 30%) and high (≥ 30%) based on previous reports [10]. TLS was defined as the presence of immune cell aggregates localized in the peritumoral stromal area. We categorized the amount of TLS according to previous reports as follows: 0 = none, no TLS formation in the area adjacent to the tumor; 1 = little, TLSs occupying an area of 1–10% of the circumference of the tumor; 2 = moderate, 11–50%; 3 = abundant, > 50% [19]. For survival analysis, we divided TLS into high (score ≥ 2) and low (score ≤ 1).

### IHC evaluation

IHC staining was performed using the following primary antibodies: PD-L1 (clone: SP142, Roche Diagnostics, Pleasanton, CA, United States of America K.K., Tokyo, Japan), CD8 (clone:4B11, Leica, Wetzlar, Germany Biosystems, Newcastle, UK), and CD20 (clone: L26, DAKO, Glostrup, Denmark). PD–L1 positivity was defined according to the manufacturer’s recommendations. The results are reported as the percent of PD-L1-stained tumor-infiltrating immune cells in the tumor stroma. A tumor was considered PD-L1 positive if ≥ 1% of tumor-infiltrating immune cells stained positive for PD-L1 (IC 1). PD-L1 positive in ≥ 5% and < 10% of tumor-infiltrating immune cells was reported as IC 2, and PD-L1 positive in ≥ 10% of tumor-infiltrating immune cells was reported as IC 3. The numbers of CD8^+^ and CD20^+^ lymphocytes were calculated as the number of positive cells/mm2 in the stroma. The stained slides were scanned using a NanoZoomer Digital Pathology System (Hamamatsu Photonics, Hamamatsu, Japan). Each specimen was reviewed at 20 × magnification, and the five areas with the greatest number of positively stained cells in the stroma were selected. Subsequently, the number of positive cells in these areas was counted using QuPath v0.2.3 (Queen’s University, Belfast, Northern Ireland) [21]. Tumors in the top 25% of positive cell counts were categorized as high, while the rest were considered low for survival analysis.

### Statistical analysis

Continuous variables reported as medians and means were compared using non–parametric and parametric tests, respectively. Categorical variables were compared using the chi-squared test. Spearman’s rank test was used to analyze the correlation between TILs, TLS, PD–L1 IC score, and CD8^+^ and CD20^+^ cells. Invasive disease-free survival (iDFS) was defined as the time from surgery to the first invasive relapse (locoregional or distant), contralateral breast cancer, or death due to any cause. The Kaplan–Meier method was used to estimate iDFS, and the log-rank test was used to compare survival between groups. Cox regression models were used to identify the prognostic value of the immune biomarkers. Clinicopathological variables associated with iDFS (*p* < 0.05) in univariable analysis were entered into a multivariable model. We evaluated the added prognostic value of immune biomarkers to the clinicopathological factors using likelihood ratio tests. All tests were two-tailed and the significance level was set at *α* = 0.05. Statistical analyses were performed using STATA (version; 15.1; StataCorp, College Station, TX, USA), GraphPad Prism ver.8.0 (GraphPad Software, San Diego, California, USA), and R software version 4.1.2 (R Core Team, Vienna, Austria).

## Results

### Patient characteristics

A total of 137 patients underwent curative surgery and did not receive chemotherapy during the study period. After excluding 12 patients for whom tumor samples were unavailable or insufficient, 125 patients were included in this analysis. Patient characteristics and their association with immune biomarkers are shown in Table 1. The median age was 68 years (range, 32–99 years). There were 124 (99.2%) patients with T1–2, 106 patients (84.8%) were node-negative, and 78 patients (62.4%) were pathological stage I. Seventy patients (56%) had histological grade 3. Histologically, 76 patients (60.8%) had invasive ductal carcinoma and 31 patients (24.8%) had apocrine carcinoma. For treatment, 67 patients (53.6%) underwent lumpectomy, and 50 patients (40%) were treated with radiotherapy.

**Table 1:** Patient characteristics and association with immune markers

	Total, *n* (%)	TILs, median (IQR)	*p*	PD–L1( +), *n* (%)	*p*	High TLS, *n* (%)	*p*	CD8, median (IQR)	*p*	CD20, median (IQR)	*p*
Age, median (range) < 65 years ≥ 65 years	68 (32–99) 48 (38.4) 77 (61.6)	10 (0–25) 10 (0–30)	0.73	11 (22.9) 25 (32.5)	0.30	12 (25.5) 15 (19.5)	0.43	900 (255–3311) 1158 (445–2818)	0.55	356 (32–2678) 303 (77–1879)	0.72
Tumor stage, T1ab ≥ T1c	33 (26.4) 92 (73.6)	10 (0–40) 10 (0–30)	0.71	9 (27.3) 27 (29.3)	0.82	6 (18.8) 21 (22.8)	0.63	965.5 (232.5–3010.5) 1082 (303.5–3064.5)	0.67	128 (13–1873.5) 461 (56–2114)	0.16
Lymph nodes status Negative Positive	106 (84.8) 19 (15.2)	10 (0–30) 10 (0–30)	0.9	29 (27.4) 7 (36.8)	0.40	25 (23.8) 2 (10.5)	0.20	955 (274–2818) 1491 (614–4040)	0.27	356 (50–1887) 275 (38–1959)	0.95
Stage I II, III	78 (62.4) 47 (37.6)	10 (0–30) 10 (0–30)	0.6	21 (26.9) 15 (31.9)	0.6	14 (18.2) 13 (27.7)	0.2	688 (235–2645) 1390 (804–4040)	0.02	123 (21–1507) 1013 (155–2678)	< 0.01
Histological grade 1–2 3	55 (44.0) 70 (56.0)	0 (0–10) 20 (0–40)	< 0.01	2 (3.6) 34 (48.6)	< 0.01	3 (5.6) 24 (34.3)	< 0.01	481.5 (216–1140) 2333.5 (814–4194)	< 0.01	98.5 (21–454) 1183 (153–3276)	< 0.01
Histology Ductal Apocrine Others	76 (60.8) 31 (24.8) 18 (14.4)	20 (0–35) 10 (0–10) 5 (0–10)	< 0.01	31 (40.8) 1 (3.2) 4 (22.2)	< 0.01	23 (30.7) 2 (6.5) 2 (11.1)	0.01	1392 (370–4194) 882 (309–1723) 529 (255–1315)	0.02	612 (92–2678) 105 (23–472) 366 (21–2813)	0.06

*TILs* tumor-infiltrating lymphocytes; *PD–L1* programmed cell death-ligand 1; *TLS* tertiary lymphoid structure; *IQR* interquartile range

The median levels [interquartile range (IQR)] of stromal TILs, CD8, and CD20 were 10% (0–30%), 1082 (295–3010.5), and 354 (45.5–1923), respectively. Thirty five patients (28%) were classified as high TILs (≥ 30%). PD-L1 in the immune cells was positive (≥ 1%) in 36 patients (28.8%). Sixty three patients (50.4%) presented with TLS (≥ 1% of the circumference of the tumor), and 57 (45.6%) had high TLS (≥ 11% of the circumference of the tumor). Increased TILs, PD-L1 positivity, CD8, CD20, and high TLS were associated with higher histological grade. Increased TILs, PD-L1 positivity, CD8, and high TLS were associated with ductal histology. A total of 29 iDFS events were observed. The median follow–up period was 77.4 months (95%CI 6.4–145.5).

### Correlation between immune biomarkers

**Fig. 1:**
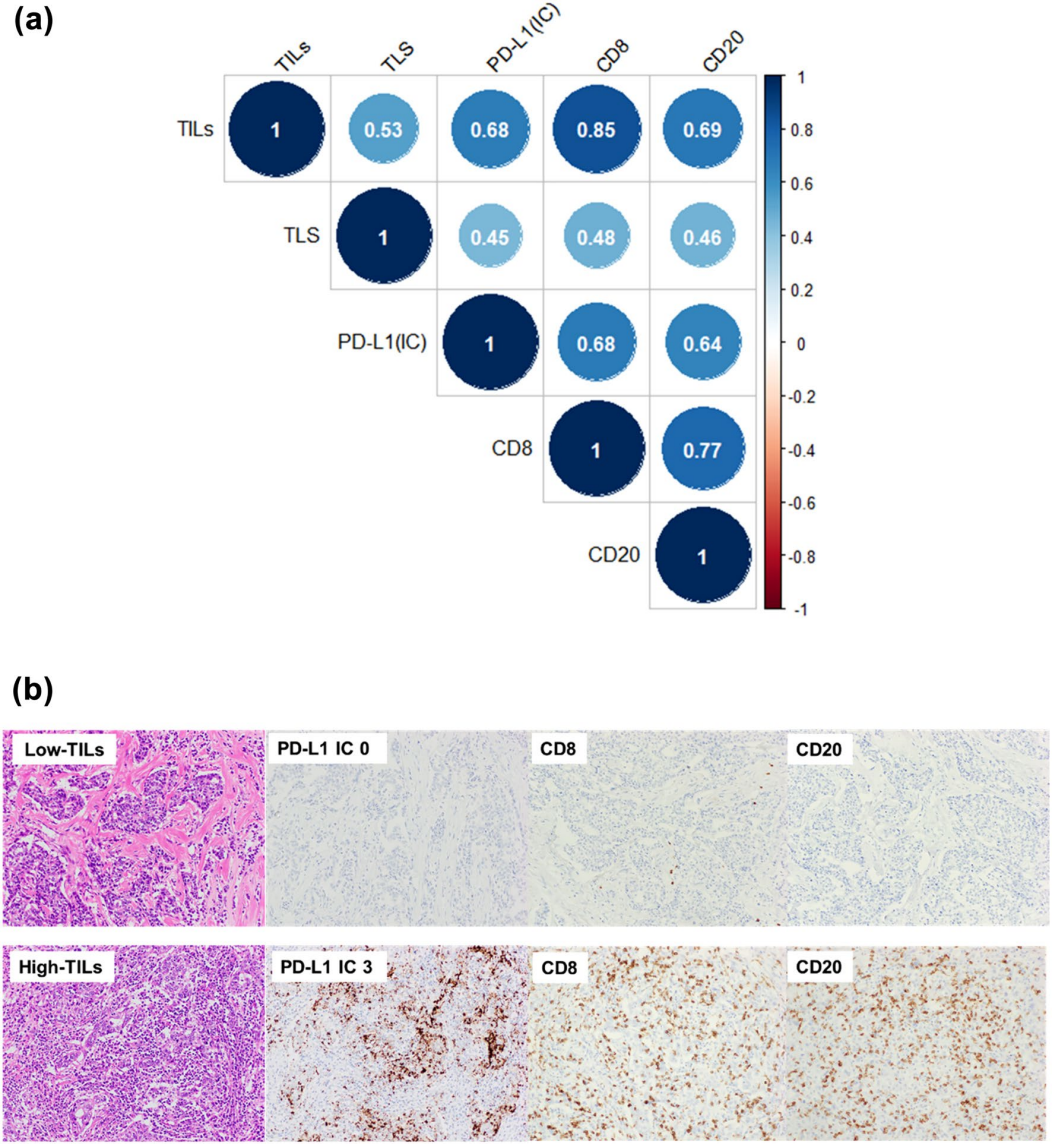
Correlation between immune biomarkers and representative images of pathology slides. **a** Spearman’s correlation coefficient between immune biomarkers. *p*-value was < 0.001 for all correlations. **b** Representative images of pathology slides show tumors with low or high TILs with the corresponding PD-L1, CD8, CD20 staining, and TLS. Abbreviations: *TILs*, tumor-infiltrating lymphocytes; *PD-L1*, programmed cell death-ligand 1; *TLS*, tertiary lymphoid structure

TILs, CD8, CD20, PD-L1, and TLS were significantly positively correlated with each other (Fig. 1a). TILs were strongly correlated with CD8 (*r* = 0.85, *p* < 0.001), CD20 (*r* = 0.69, *p* < 0.001), and PD-L1 IC score (*r* = 0.68, *p* < 0001). TLS were moderately associated with TILs (*r* = 0.53, *p* < 0.001), PD-L1 (*r* = 0.45, *p* < 0.001), CD8 (*r* = 0.48, *p* < 0.001), and CD20 (*r* = 0.46, *p* < 0.001). Representative pictures of PD-L1, CD8, and CD20 staining in tumors with low or high TILs are shown in Fig. 1b. Supplementary Fig. 1 shows representative images of cases with absent and abundant TLS.

### Association of immune biomarkers and survival in TNBC

In univariable analysis, each 10% increment in TILs and high TILs (≥ 30%) was not significantly associated with better iDFS [hazard ratio (HR) 0.99, 95% confidence interval (CI) 0.97–1.01, *p* = 0.28 for 10% increment; HR 0.54, 95%CI 0.20–1.41, *p* = 0.21 for TILs ≥ 30%]. High CD8 (top 25%), high CD20 (top 25%), PD-L1 positivity, and high TLS were also not associated with iDFS (HR 0.83, 95%CI 0.63–1.08, *p* = 0.16 for high CD8; HR 0.91, 95%CI 0.71–1.16, *p* = 0.44 for high CD20; HR 1.65, 95%CI 0.76–3.5, *p* = 0.2 for PD–L1; HR 1.17, 95%CI 0.50–2.76, *p* = 0.72 for TLS). Stage and histological grade were significantly associated with iDFS and were included in the multivariable model as clinicopathological variables (Supplementary Table 1). In multivariable analysis adjusted for clinicopathological factors, only high CD8 was significantly associated with better iDFS (HR 0.74, 95%CI 0.56–0.97, *p* = 0.03). Increased TILs showed a trend toward a significant association with better iDFS (HR 0.98, 95%CI 0.95–1.00, *p* = 0.07 for 10% increment; HR 0.38, 95%CI 0.14–1.02, *p* = 0.05 for TILs ≥ 30%). High CD20, PD-L1 positivity, and TLS were not associated with iDFS (Supplementary Table 2).

We then evaluated the prognostic impact of each immune biomarker, in addition to clinicopathological factors. TILs, PD-L1, TLS, and CD20 did not provide significant additional prognostic information when combined with clinicopathological factors. Only CD8 conferred significant prognostic information when combined with clinicopathological factors (likelihood test, χ^2^ = 6.28, *p* = 0.04) (Table 2). Given that TILs are established prognostic factors for early-stage TNBC, we evaluated the improvement in model fit when other immune biomarkers were added to the clinicopathological factors and TILs. Adding PD-L1, TLS, CD8, or CD20 did not significantly improve the model fit. However, adding both PD-L1 and CD8 significantly improved the prognostic model (likelihood test χ^2^ = 9.24, *p* = 0.01) (Table 2). In a Cox regression model including TILs, CD8, PD-L1, and clinicopathological factors, high CD8 was significantly associated with better prognosis (HR 0.69, 95%CI 0.48–0.98, *p* = 0.04), and PD-L1 positivity was significantly associated with worse prognosis (HR 4.33, 95%CI 1.57–11.99, *p* = 0.005) (Table 3).

**Table 2:** Additional prognostic value of immune biomarkers in multivariable Cox regression models

	Likelihood ratio test Chi–squared value	*p*-value
CP + TILs (≥ 30%) vs. CP	4.33	0.11
CP + PD-L1 vs. CP	0.22	0.90
CP + TLS vs. CP	0.55	0.76
CP + CD8 vs. CP	6.28	0.04
CP + CD20 vs. CP	2.93	0.23
CP + TILs (≥ 30%) + PD-L1 vs. CP + TILs (≥ 30%)	4.57	0.10
CP + TILs (≥ 30%) + TLS vs. CP + TILs (≥ 30%)	0.13	0.93
CP + TILs (≥ 30%) + CD8 vs. CP + TILs (≥ 30%)	2.07	0.36
CP + TILs (≥ 30%) + CD20 vs. CP + TILs (≥ 30%)	0.77	0.68
CP + TILs (≥ 30%) + PD-L1 + CD8 vs. CP + TILs (≥ 30%)	9.24	0.01

*CP* clinicopathological factors (stage, histological grade); *TILs* tumor-infiltrating lymphocytes; *PD*-*L1* programmed cell death-ligand 1; *TLS* tertiary lymphoid structure

**Table 3:** Multivariable Cox regression model for iDFS

	Multivariable		
	HR	95% CI	*p*-value
TILs (≥ 30% vs. < 30%)	0.42	0.11–1.70`	0.23
CD8 (high vs. low)	0.69	0.48–0.98	0.04
PD-L1 (positive vs. negative)	4.33	1.57–11.99	0.005
Stage (II, III vs. I)	1.74	0.77–3.93	0.18
Histologic grade (3 vs. 1–2)	2.09	0.82–5.35	0.12

*iDFS* invasive disease-free survival; *TILs* tumor-infiltrating lymphocytes; *PD*-*L1* programmed cell death-ligand 1; *TLS* tertiary lymphoid structure; *HR* hazard ratio; *CI* confidence interval

### Survival probabilities by CD8^+^ TILs and PD-L1 expression

**Fig. 2:**
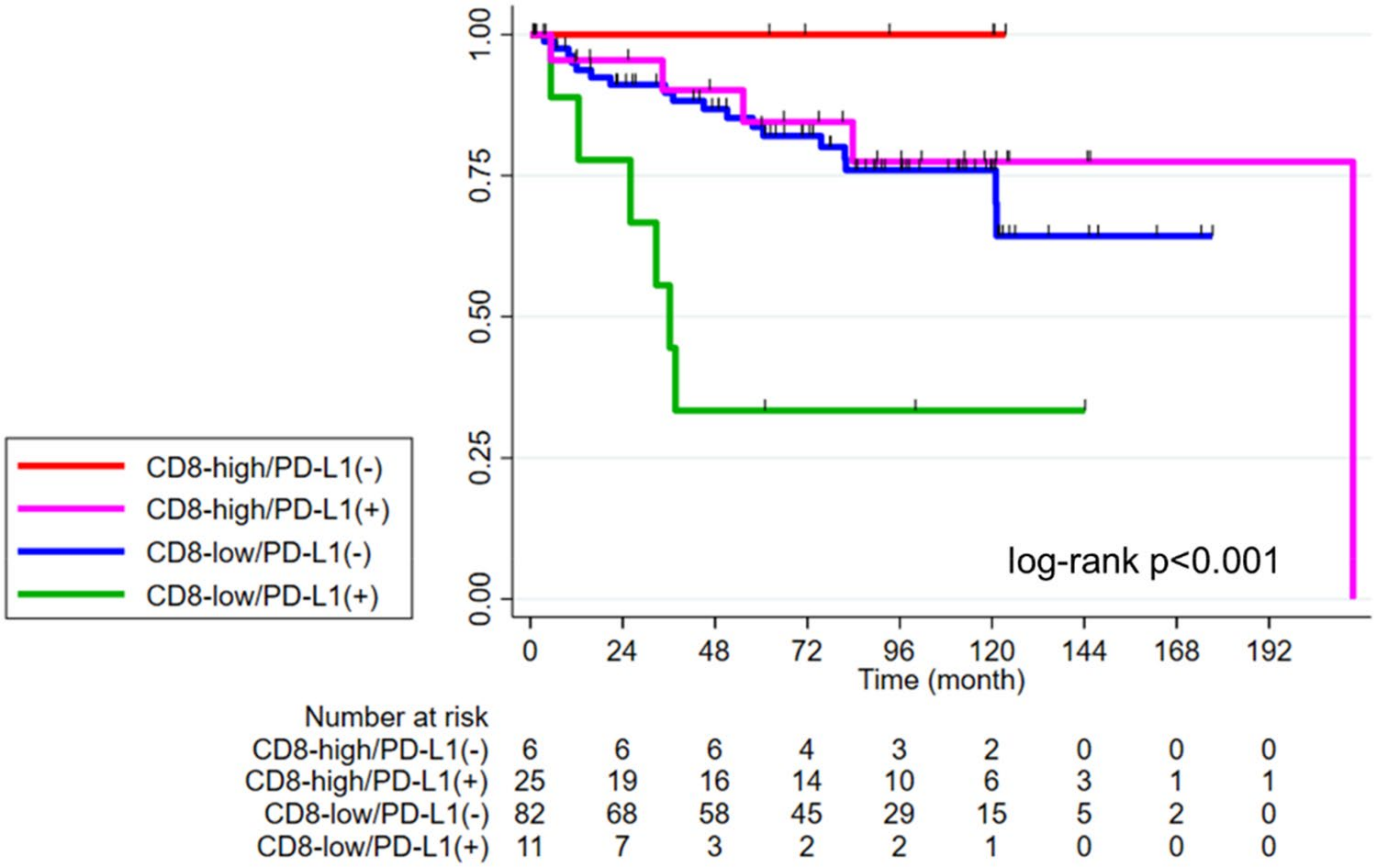
Kaplan–Meier curves for iDFS by CD8 and PD*-*L1 expression. iDFS in patients with high CD8/PD*-*L1(−) (red) vs. low CD8/PD-L1(−) (blue) vs. low CD8/PD-L1( +) (green) vs. high CD8/PD-L1( +) (purple). Abbreviations: *PD-L1*, programmed cell death-ligand 1; *iDFS*, invasive disease-free survival

The 5 year iDFS was 80.7% (95%CI 71.9–87.0) in the total population. The 5 year iDFS was 85.6% (95%CI, 66–94.3) in the high TIL group compared to 79.0% (95% CI 68.3–86.5) in the low TIL group (log-rank *p* = 0.2). The survival curves for iDFS according to other immune biomarkers are shown in Supplementary Fig. 2. We then compared iDFS in four immune subtypes according to CD8 (high vs. low) and PD-L1 expression (positive vs. negative). Patients with high CD8/PD-L1(−) tumors had the most favorable prognosis (5 year iDFS 100%), while patients with low CD8/PD-L1( +) tumors had the worst prognosis (5 year iDFS 33.3%, 95%CI 7.8–62.3). Patients with high CD8/PD-L1( +) tumors and low CD8/PD-L1(−) tumors had intermediate prognosis (5 year iDFS 84.5%, 95%CI 59.1–94.8; 5 year iDFS 83.9%, 95%CI 73.2–90.5, respectively; log-rank *p* < 0.001) (Fig. 2).

### Difference in CD20 and TLS levels between four immune subtypes based on CD8 and PD-L1

**Fig. 3:**
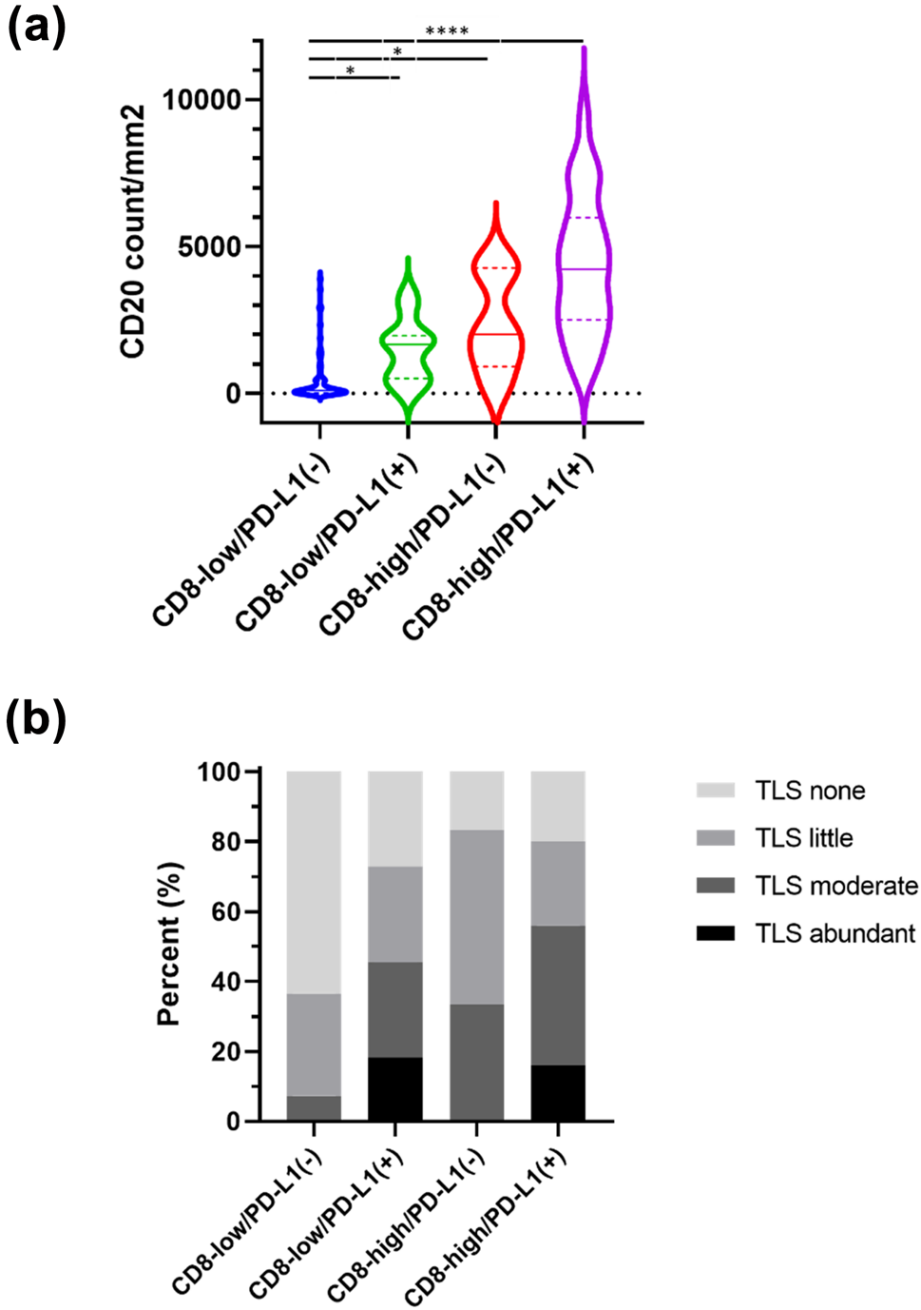
CD20 positive cells and TLS between four immune subtypes based on CD8 and PD-L1. Violin plots of CD 20 positive cells (**a**) in patients with low CD8/PD-L1(−) (blue), high CD8/PD-L1(−) (red), low CD8/PD-L1( +) (green), and high CD8/PD-L1( +) (purple). The bars represent the first, median, and third quartile values. *p < 0.05, ****p < 0.0001. **b** Association between TLS amount and immune subtypes. Abbreviations: *PD*-*L1*, programmed cell death-ligand 1; *TLS*, tertiary lymphoid structure

We explored the association of CD20 and TLS status with four immune subtypes. CD20 was significantly lower in patients with low CD8/PD-L1(−) tumors than in other subtypes [median (IQR) CD20 for low CD8/ PD-L1(−) vs. low CD8/PD-L1( +) vs. high CD8/PD-L1(−) vs. high CD8/PD-L1( +); 99.5 (17.5–433) vs. 1674 (512–1959) vs. 2003 (910–4276) vs. 4230 (2503–5983), *p* < 0.001) (Fig. 3a]. After excluding low CD8/PD-L1(−) tumors, low CD8/PD-L1( +) tumors had lower CD20^+^ lymphocyte counts than high CD8/PD-L1( +) tumors [*p* < 0.001, Dunn test *p* < 0.001 between low CD8/PD-L1( +) vs. high CD8/PD-L1( +)]. However, CD20 + lymphocytes did not differ significantly between low CD8/PD-L1( +) and high CD8/PD-L1(−) tumors (*p* = 0.99). The proportion of high TLS differed between immune subtypes: 7.3%, 45.5%, 33.3%, and 56.0% in low CD8/PD-L1(−), low CD8/PD-L1( +), high CD8/PD-L1(−) and high CD8/PD-L1( +) groups, respectively (*p* < 0.001) (Fig. 3b).

## Discussion

In this study, we evaluated the prognostic value of CD8^+^ and CD20^+^ lymphocytes, PD-L1, and TLS in addition to TILs in patients with TNBC not treated with adjuvant chemotherapy. The combination of CD8 and PD-L1 significantly improved the prognostic model using standard clinicopathological factors and TILs. Four immune subtypes, based on CD8 and PD-L1 can stratify iDFS. Although previous studies have assessed the relationship between these immune biomarkers and prognosis for early-stage TNBC [14, 15, 19, 22], few have assessed their prognostic value in TNBC without adjuvant chemotherapy.

Three retrospective studies showed that increased TILs were significantly associated with favorable outcomes in TNBC patients who did not receive chemotherapy [10, 23, 24]. Our study showed a trend toward a significant association between increased TILs and better iDFS, but this was not significantly different. This result was attributed to the small sample size of the study. A previous study also required a pooled analysis of 479 patients from four independent cohorts to clarify the significant association between TILs and iDFS [10]. In contrast, high CD8 levels were significantly associated with favorable outcomes, in agreement with previous findings [14, 25–27]. TILs in breast tumors comprise immune cell subpopulations, including T cells, B cells, macrophages, and natural killer cells [5]. CD8^+^ lymphocytes are major components of tumor-specific adaptive immune responses and may reflect antitumor immunity more specifically than the global evaluation of TILs.

Adding both CD8 and PD-L1 to clinicopathological factors and TILs significantly improved the prognostic model. Previous studies also showed that combined PD-L1 to TILs provide prognostic information for TNBC treated with standard chemotherapy [14, 22]. In our model, PD-L1 expression in the immune cells was associated with poor iDFS. Although a meta-analysis of eight retrospective studies showed that PD-L1 expression in the immune cells was associated with better iDFS in TNBC [28], it is difficult to compare because of the different antibodies used, positive cutoff values, and materials. Carter et al.[17] evaluated the association between PD-L1 expression in whole tumor sections using SP142 antibody, as we did in this study, and the prognosis of 498 cases of non–metastatic TNBC. They also showed PD-L1 expression was associated with improved iDFS [17]. The following two reasons may explain the disagreement between our findings and those of previous studies: First, PD-L1 was correlated with TILs, which is known as a strong prognostic factor, which may be associated with a better prognosis. Previous studies showed that PD-L1 expression moderately correlates with TILs in TNBC (*r* = 0.45–0.59) [8, 27, 29, 30]. We also confirmed a positive correlation between PD–L1 and TILs (*r* = 0.68). Second, PD-L1 expression is a predictive marker of response to standard chemotherapy. In clinical trials that incorporated anti-PD-L1 antibody to neoadjuvant chemotherapy for TNBC (KEYNOTE–522, IMpassion031, and NeoTRIPaPDL1), the absolute pathological complete response was 15–20% higher in PD-L1 positive tumors than PD-L1 negative tumors in the standard chemotherapy arm [31–33]. The higher efficacy of perioperative chemotherapy may be associated with the better prognosis of PD-L1 positive tumors in previous studies.

Four immune subtypes based on CD8 and PD-L1 expression can significantly stratify the prognosis. The high CD8/PD-L1(−) group had the most favorable prognosis and the low CD8/PD-L1( +) group had the worst prognosis. International guidelines strongly recommend perioperative chemotherapy for early-stage TNBC ≥ T1c or positive lymph nodes [3, 34]. While de-escalation of perioperative chemotherapy for patients at low clinical or genomic risk is now possible for ER-positive and HER2-positive breast cancer [35–37], this approach has not been possible for TNBC. Early-stage TNBC with high CD8/PD-L1(−), which accounts for 4.8% of cases, has excellent prognosis without chemotherapy. The evaluation of PD-L1 and CD8, in addition to TILs, may more accurately identify populations for whom chemotherapy can be safely omitted. In systemically untreated early-stage TNBC, the 5 year iDFS in the high TIL group was approximately 80% [10, 23, 24], which is insufficient to consider omitting adjuvant chemotherapy. Our findings showed that patients with PD-L1( +) tumors had a worse prognosis than those with PD-L1(−) even at high-CD8 levels. It may not be appropriate to consider omitting chemotherapy based on high TILs alone. PD-L1 expression on immune cells is upregulated by inflammatory cytokines, particularly interferon γ, released by TILs [38, 39]. This adaptive immune resistance suppresses local TILs function and may be associated with poor prognosis. Patients with low CD8/PD-L1( +) status may be more likely to relapse, even at low clinical risk. Standard chemotherapy and PD-1/PD-L1 inhibition may not be sufficient in patients with low CD8/PD-L1( +) tumors. These patients may have a defect in earlier steps in the cancer immunity cycle, and combinational immunotherapy including anti-OX40, anti-CTLA4, or anti-angiogenic with PD-1/L1 inhibition may be required to promote tumor immune cell infiltration and improve prognosis [40, 41]. In the future, perioperative treatment should be stratified according to the individuals tumor immune microenvironment. Furthermore, it is desirable to examine whether adjuvant chemotherapy can be omitted in clinically low-risk and high CD8/PD-L1(−) TNBC, in prospective trials.

CD20^+^ lymphocytes and TLS were not significantly associated with better prognosis, either themselves or in combination with TILs. Although CD20^+^ lymphocytes and TLS have been associated with a better prognosis in TNBC [15, 19, 42, 43], they may not be prognostic factors in TNBCs not receiving chemotherapy. Alternatively, the evaluation method of the TLS could have affected the discrepancy in the results. We identified TLS with H&E staining alone; however, it may be less accurate in detecting TLS than IHC staining for CD3, CD45, and MECA79 [19, 44, 45].

We also demonstrated that the tumor immune microenvironment differed by histological type. Patients with apocrine carcinoma, a rare type of primary breast cancer, had lower TILs, CD8 levels, and PD-L1 expression than those with ductal carcinoma. Sun et al. [46] showed that the median TILs and PD-L1 expressions were 20% and 11.7% in 18 triple-negative apocrine carcinoma cases, respectively. Moreover, the loss of MHC class I expression was observed in 78% of triple-negative apocrine carcinoma cases [47]. Approximately 90% of apocrine carcinomas involve genetic abnormalities in the PI3K/mTOR pathway, and this may suppress T-cell infiltration [46, 48].

Our study has some limitations. This was a retrospective study conducted at a single institution with a small sample size. Patients with early-stage TNBC who are not treated with chemotherapy are rare, and our study was one of the largest studies from a single institution. There was potential selection bias because the reasons for omitting chemotherapy varied for each patient. The high proportion of older patients and special histological types in our study population may not be extrapolated to the general population. However, the 85% 5 year iDFS in the high TIL group of our cohort was comparable to that of previous studies [10, 23, 24], supporting the acceptability of our results. Further validation in a larger cohort is required to confirm our findings. The strengths of our study are that it evaluated the prognostic value of multiple immune biomarkers concurrently with TILs, and that its prognostic value was not influenced by chemotherapy.

In conclusion, CD8 and PD-L1 expression in immune cells, but not CD20 and TLS, provides significant prognostic value beyond TILs in patients with early-stage TNBC not treated with chemotherapy. Patients with high CD8/PD-L1(−) tumors are associated with excellent prognosis, while low CD8/PD-L1( +) tumors are associated with poor prognosis. Further research is warranted to optimize perioperative treatments based on the individual tumor microenvironment.

## Supplementary Information

Below is the link to the electronic supplementary material.

Supplementary file1 (DOCX 1623 kb)

## Data Availability

The data analyzed in this study are available from the corresponding author upon reasonable request.

## Abbreviations

CI: Confidence interval
ER: Estrogen receptor
FISH: Fluorescence in situ hybridization
H&E: Hematoxylin and eosin
HER2: Human epidermal growth factor receptor 2
HR: Hazard ratio; iDFS, invasive disease-free survival
IHC: Immunohistochemical
IQR: Interquartile range
PD-1: Programmed cell death receptor 1
PD-L1: Programmed cell death-ligand 1
PR: Progesterone receptors
TILs: Tumor-infiltrating lymphocytes
TLS: Tertiary lymphoid structures
TNBC: Triple-negative breast cancer
