# Supplementary material for: Integrative prognostic analysis of tumor–infiltrating lymphocytes, CD8, CD20, programmed cell death-ligand 1, and tertiary lymphoid structures in patients with early-stage triple-negative breast cancer who did not receive adjuvant chemotherapy
Source: Breast Cancer Res Treat. 2022 Nov 16;197(2):287–97. doi: 10.1007/s10549-022-06787-x (PMC9823028; doi:10.1007/s10549-022-06787-x)
Supplement: Supplementary file 1 — Supplementary file1 (DOCX 1623 kb) [file 10549_2022_6787_MOESM1_ESM.docx]

**SUPPLEMENTAL MATERIALS**

**Supplementary Table 1. Univariable Cox regression analysis of each clinicopathological factor and immune biomarker for iDFS**

|  | HR | 95% CI | P value |
| --- | --- | --- | --- |
| TILs (≥30% vs. <30%) | 0.54 | 0.20–1.41 | 0.21 |
| TILs (10% increment) | 0.99 | 0.97–1.01 | 0.28 |
| PD–L1 (positive vs. negative) | 1.65 | 0.76–3.58 | 0.20 |
| CD8 (high vs. low) | 0.83 | 0.63–1.08 | 0.16 |
| CD20 (high vs. low) | 0.91 | 0.71–1.16 | 0.44 |
| TLS (≥10% vs. <10%) | 1.17 | 0.50–2.76 | 0.72 |
| age (≥65 vs. <65) | 1.54 | 0.68–3.50 | 0.30 |
| stage (II, III vs. I) | 2.17 | 1.03–4.57 | 0.04 |
| histology (IDC vs. special) | 2.34 | 0.99–5.5 | 0.05 |
| histological grade (3 vs. 1–2) | 2.35 | 1.03–5.35 | 0.04 |
| adjuvant RT (yes vs. no) | 0.68 | 0.31–1.47 | 0.32 |

Abbreviations: TILs, tumor–infiltrating lymphocytes; PD–L1, programmed cell death- ligand 1, TLS, tertiary lymphoid structure; iDFS, invasive disease–free survival; HR, hazard ratio; CI, confidence interval; IDC, invasive-ductal carcinoma

**Supplementary Table 2. Association between each immune biomarker and iDFS adjusted by stage and histological grade**

|  | HR | 95% CI | P value |
| --- | --- | --- | --- |
| TILs (≥30% vs. <30%) | 0.38 | 0.14–1.02 | 0.05 |
| TILs (10% increment) | 0.98 | 0.95–1.00 | 0.07 |
| PD–L1 (positive vs. negative) | 1.23 | 0.52–2.89 | 0.64 |
| CD8 (high vs. low) | 0.74 | 0.56–0.97 | 0.03 |
| CD20 (high vs. low) | 0.83 | 0.64–1.06 | 0.12 |
| TLS (≥10% vs. <10%) | 0.77 | 0.31–1.91 | 0.58 |

Abbreviations: TILs, tumor–infiltrating lymphocytes; PD–L1, programmed cell death- ligand 1, TLS, tertiary lymphoid structure; iDFS, invasive disease–free survival; HR, hazard ratio; CI, confidence interval


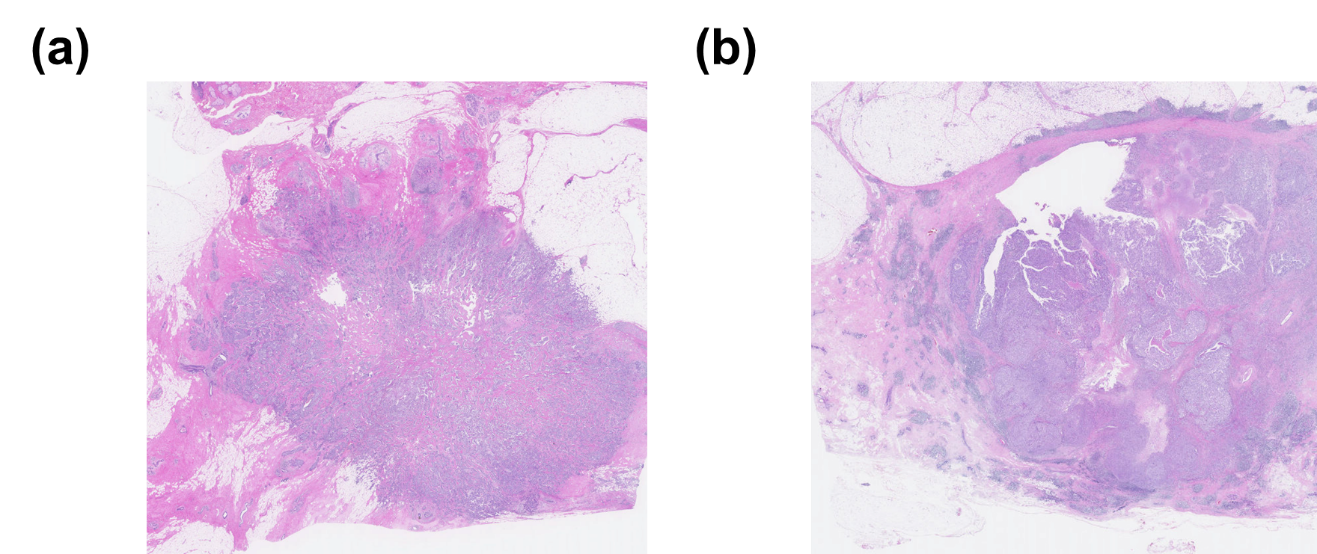


**Supplementary Fig. 1 Representative images of TLS** Representative images of cases with absent (**a**) and abundant (**b**) TLS in the peritumoral stromal area (scanning magnification).

Abbreviation: TLS, tertiary lymphoid structure


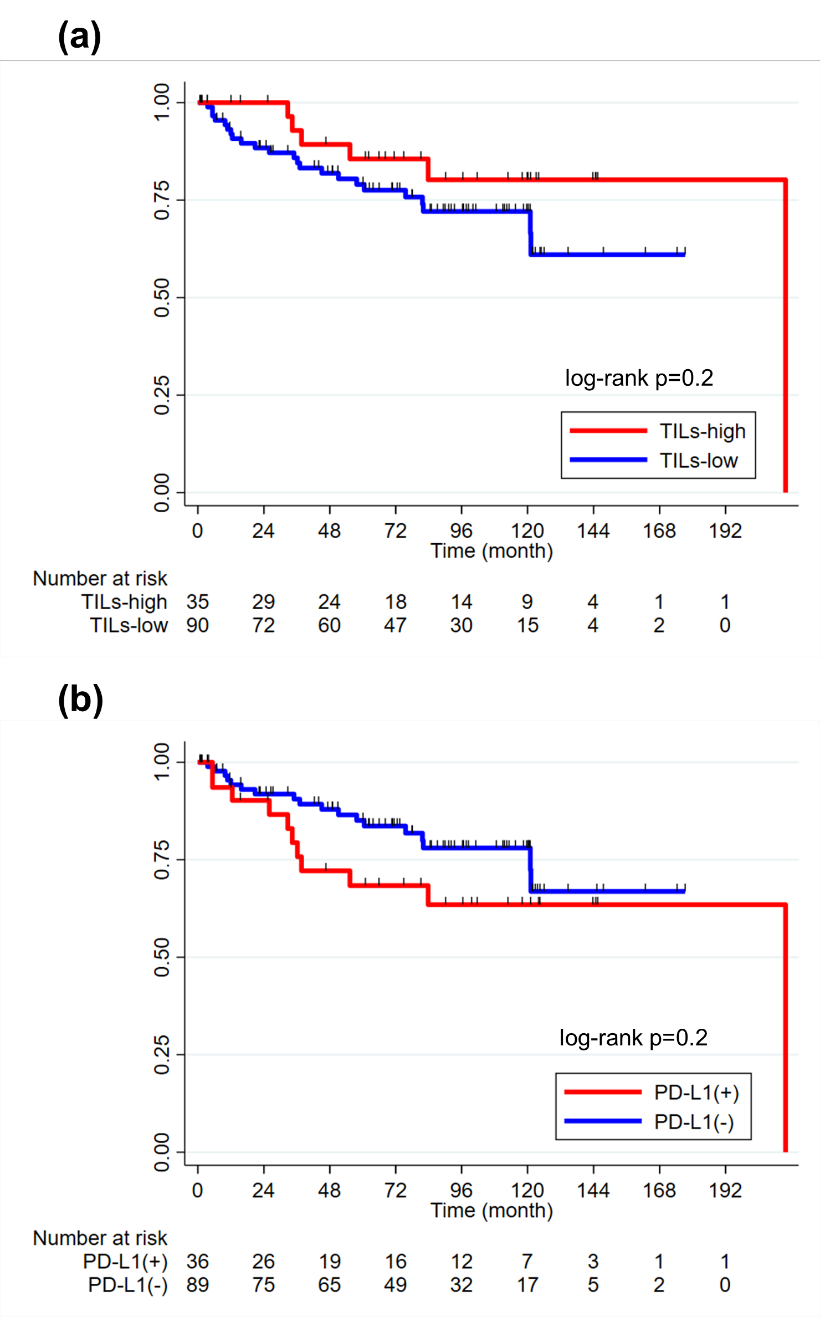


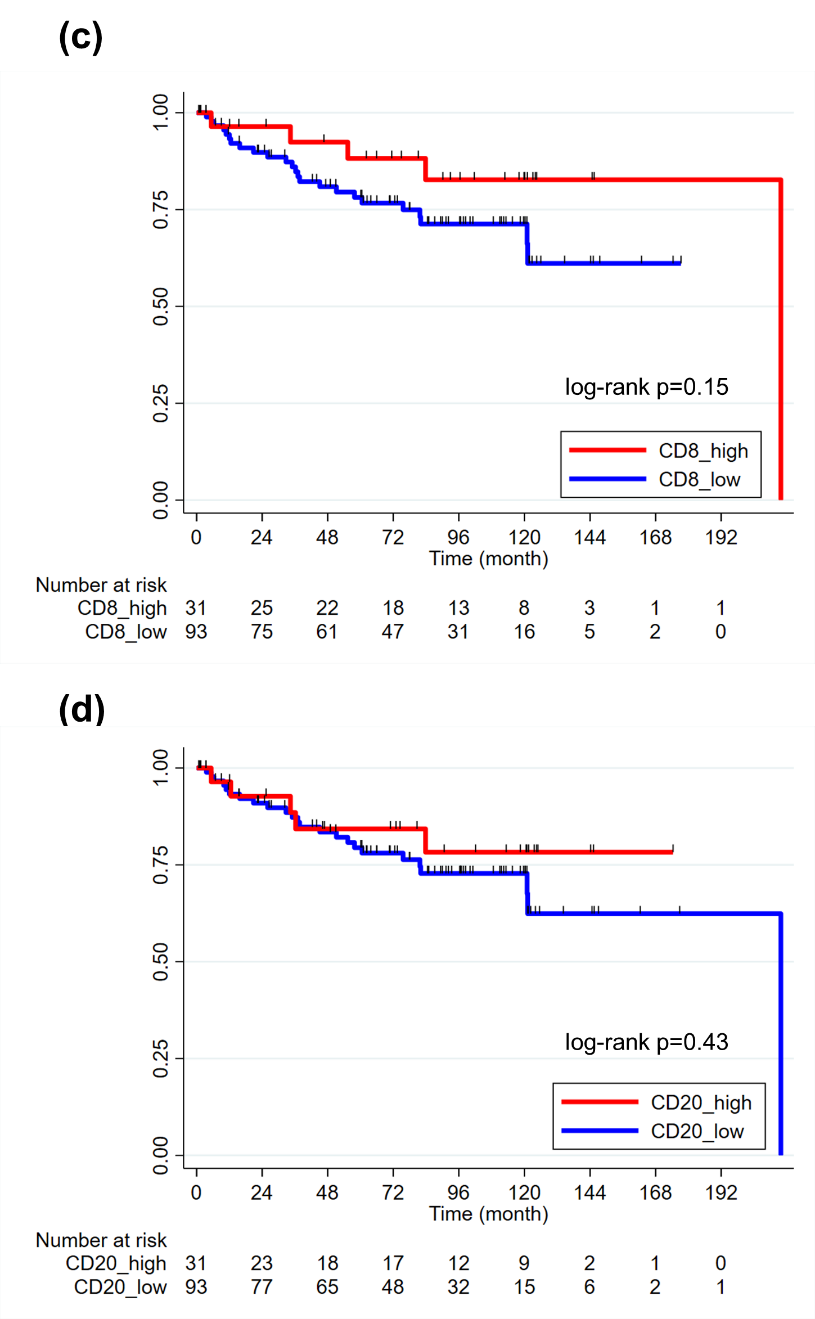


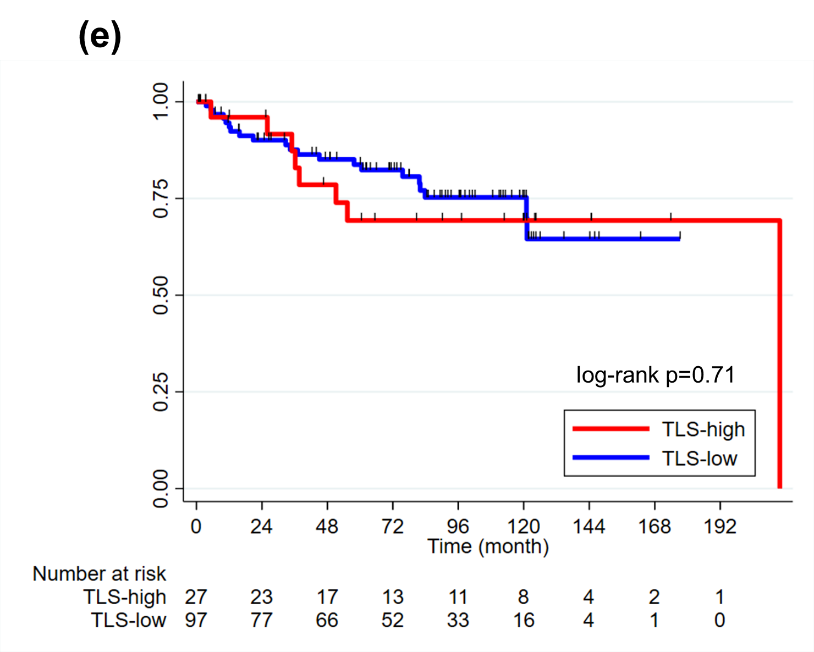


**Supplementary Fig. 2 Kaplan–Meier curves for iDFS according to immune biomarkers** iDFS in patients with high TILs (red) vs. low TILs (blue) (**a**), PD–L1(+) (red) vs. PD–L1(–) (blue) (**b**), high CD8 (red) vs. low CD8 (blue) (**c**), high CD20 (red) vs. low CD20 (blue) (**d**) and high TLS (red) vs. low TLS (blue) (**e**).

Abbreviations: iDFS, invasive disease–free survival; TILs, tumor–infiltrating lymphocytes; PD–L1, programmed cell death- ligand 1; TLS, tertiary lymphoid structure
